# The efficacy and safety of Chinese herbal compound or combined with western medicine for pediatric adenoidal hypertrophy: A protocol for systematic review and meta-analysis

**DOI:** 10.1097/MD.0000000000022023

**Published:** 2020-09-04

**Authors:** Penglin Wang, Weidong Kong, Yanchun Shan

**Affiliations:** a Department of Orthodontics, School of Stomatology, Jinan University; b Department of Orthodontics, First Affiliated Hospital of Jinan University; c Department of Paediatrics, School of Nursing, Jinan University, Guangzhou, China.

**Keywords:** adenoid hypertrophy, children, system evaluation, traditional Chinese medicine

## Abstract

**Background:**

Traditional Chinese medicine (TCM) or combined with western medicine in the treatment of pediatric adenoidal hypertrophy has been widely used in clinical practice, but the overall efficacy and safety is still unclear. This paper aims to evaluate the efficacy and safety analysis of TCM or combined with western medicine for pediatric adenoidal hypertrophy.

**Methods:**

PubMed, EMbase, Cochrane Library, Web of Science, China National Knowledge Infrastructure (CNKI), WanFang, the Chongqing VIP Chinese Science and Technology Periodical Database, and China biomedical literature database (CBM) were searched for randomized controlled trials of TCM or combined with western medicine for pediatric adenoidal hypertrophy from the date of establishment to July 2020, and Baidu Scholar, Google Scholar, International Clinical Trials Registry Platform (ICTRP), and Chinese Clinical Trials Registry (ChiCTR) were searched for unpublished grey literature. Two researchers independently applied RevMan 5.3 software for data extraction and risk assessment of bias.

**Results:**

The effectiveness and safety of TCM or combined with western medicine for pediatric adenoidal hypertrophy is evaluated by means of the Adenoid (A) /(Nasopharyngeal (N) ratio, clinical efficacy, integral score of TCM syndromes, clinical single symptom score, disease specific quality of life for children with obstructive sleep apnea 18 items survey (OSA-18), Interleukin 4 (IL-4) and adverse reaction incidence.

**Conclusion:**

This study will provide theoretical support for the clinical application of TCM or combined with western medicine for pediatric adenoidal hypertrophy.

**OSF Registration number:**

DOI 10.17605/OSF.IO/J76AG.

## 1 Introduction

Pediatric adenoidal hypertrophy is a common respiratory disease in pediatrics. It is usually caused by infection and other inflammatory stimuli, resulting in pathological hyperplasia and hypertrophy of adenoids in children. Clinical symptoms are often accompanied by changes in breathing patterns such as nasal congestion, snoring, mouth opening, etc. Resulting in malformation of maxillofacial development and appearance of adenoid face in children, and complications turn up: obstructive sleep apnea Syndrome (OSAS), sudden infant death syndrome (SIDS) and so on.^[1]^ The incidence of this disease is 9.9% to 29.9%, and increased year by year. The etiology and pathogenesis of this disease are not clear, and it is believed that it is closely related to infection, allergy, immunity, and other factors. Besides, adenoid hypertrophy has adverse effects on the jaw development of children and affects the abnormal occlusal relationship of anterior incisors. Therefore, children must first treat adenoid hypertrophy before orthodontics in stomatology department. At present, surgery and drugs are the main ways to treat this disease. The operation is effective quickly, the operation requires general anesthesia, the children have poor cooperation and tolerance, and there are risks such as postoperative complications. Most children and their parents refuse surgery and prefer drug therapy. Studies have shown that the application of nasal glucocorticoids can down-regulate the activity of glandular lymphocytes, regulate the microenvironment of glandular flora, inhibit nasopharyngitis and dysplasia of glandular tissues, and even shrink the hyperplasia of glandular tissues.^[2]^ Montelukast is a cysteinyl LT receptors 1 (Cys LTR1) antagonist. Leukotriene C4 (LTC4), Leukotriene D4 (LTD4), Leukotriene E4 (LTE4) Combined with Cys LTR1, it caused glandular tissue edema, tissue hyperplasia, increased vascular permeability, inflammatory cell infiltration, and induced a series of pathological changes of the upper respiratory tract. Clinical application of Montelukast can effectively improve the ventilation function and abnormal symptoms of sleep breathing in children.^[3]^

Traditional Chinese medicine treatment mainly focuses on promoting blood circulation, dispersing phlegm and strengthening spleen. The clinical protocol is usually single use of Traditional Chinese medicine or combined use of western medicine. Traditional Chinese medicine treatment is not the same, because of the lack of systematic evaluation and different clinical trial research design and efficacy, results are mixed. This study intends to conduct a meta-analysis to systematically evaluate the efficacy and safety of TCM or combined with western medicine for pediatric adenoidal hypertrophy.

## 2 Methods

### 2.1 Protocol register

This protocol of systematic review and meta-analysis has been drafted under the guidance of the preferred reporting items for systematic reviews and meta-analyses protocols (PRISMA-P). Moreover, it has been registered on open science framework (OSF) (Registration number: DOI 10.17605/OSF.IO/J76AG).

### 2.2 Ethics

Ethical approval is not required because there is no patient recruitment and personal information collection, and the data included in our study are derived from published literature.

### 2.3 Inclusion criteria for research programmes

#### 2.3.1 Type of studies

Including randomized controlled trials of Chinese herbal compound alone or combined with Western medicine for pediatric adenoidal hypertrophy. Languages are English and Chinese only.

#### 2.3.2 Study object

All the included cases are in line with the “Consensus of TCM Diagnosis and Treatment Experts on Sleep breathing Disorder caused by Pediatric Adenoidal Hypertrophy,”^[4]^ regardless of nationality, race, age, gender, and course of disease of the patients.

#### 2.3.3 Intervention type

The control group adopted conventional western medicine treatment, including nasal hormone drugs (such as Inhaled Corticosteroids (ICS)), anti-leukotriene drugs (such as oral montelukast sodium (OM)), with unlimited western medicine types. The observation group was treated with Chinese medicine alone or combined with Chinese medicine on the basis of the control group.

#### 2.3.4 Observational index

(1) Primary outcome: the Adenoid (A)/(Nasopharyngeal (N) ratio;
(2) Secondary outcomes: (1) Clinical efficacy score; (2) Integral score of TCM syndromes; (3) Clinical single symptom score; (4) Disease specific quality of life for children with obstructive sleep apnea 18 items survey (OSA-18) score; (5) Interleukin 4 (IL-4); (6) Adverse reaction incidence.

### 2.4 Exclusion criteria

(1) The literature was published as abstract and conference, and the data of the article could not be extracted by contacting the author;
(2) Eliminate the repeatedly published articles and input the research with the most complete information;
(3) The original data is incomplete or has errors;
(4) There are obvious mistakes in the randomization of the articles.

### 2.5 Search strategy

Retrieval was conducted in PubMed, EMbase, Cochrane Library, Web of Science, China National Knowledge Infrastructure (CNKI), WanFang, the Chongqing VIP Chinese Science and Technology Periodical Database, and China biomedical literature database (CBM), for randomized controlled trials of TCM or combined with western medicine for pediatric adenoidal hypertrophy. Retrieval time: establishment of database until July 2020. At the same time, search Baidu, Google Scholar, International Clinical Trials Registry Platform (ICTRP), and Chinese Clinical Trials Registry (ChiCTR) to get unpublished grey literature. The retrieval words are: Traditional Chinese medicine, Chinese herbal compound, Adenoidal hypertrophy, etc. PubMed retrieval strategies are shown in Table 1.

**Table 1:**
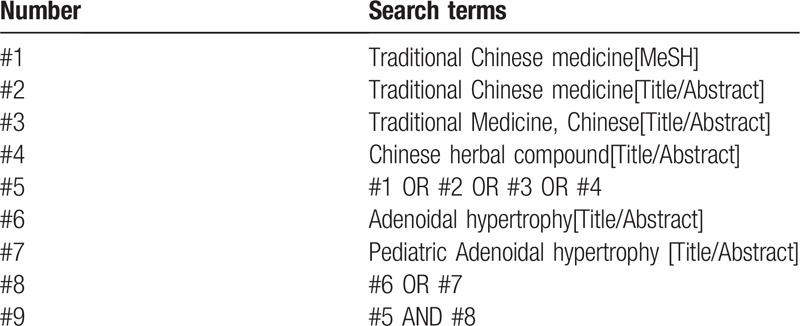
Search strategy in PubMed database.

### 2.6 Data extraction principle

**Figure 1:**
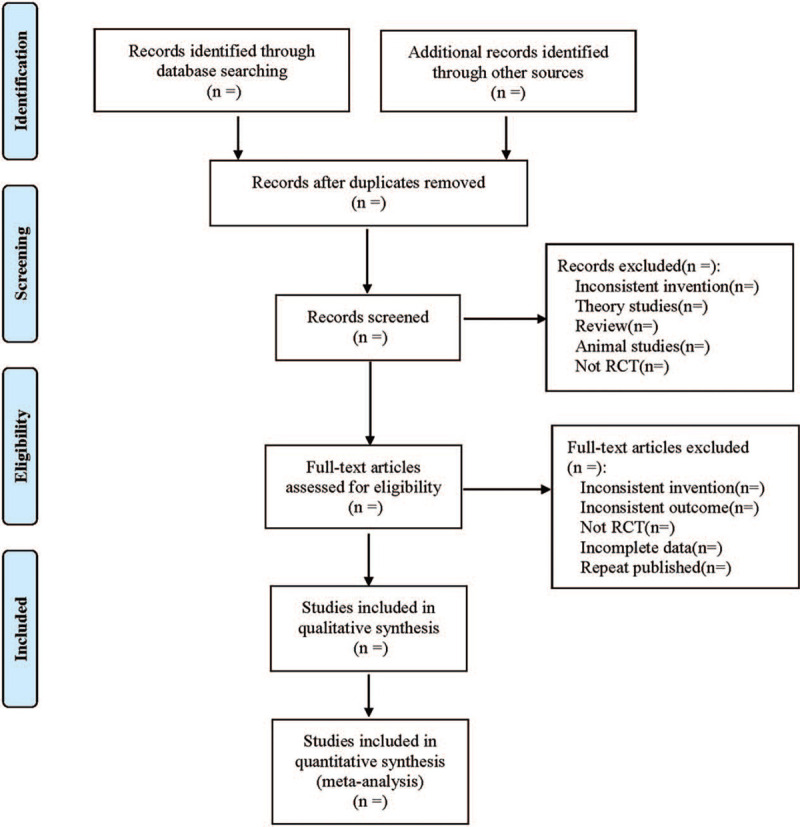
Flow diagram.

Referring to the literature quality evaluation criteria of the Cochrane Collaboration Handbook of Systematic Reviewers version 5.0, the two researchers independently screened, extracted and included the literature based on the above inclusion and exclusion criteria. Endnote X7 was used to manage the literature, and the reasons for the exclusion were recorded, conflicting papers should be discussed with a third researcher. Excel 2019 literature information database was established to extract data including:

(1) Clinical studies (title, first author, publication date, sample size, sex ratio, average age, average course of disease);
(2) Intervention measures (name, dose and course of treatment of western medicine in the control group; the traditional Chinese medicine, dosage and course of treatment used in the treatment group);
(3) Risk bias assessment factors in randomized controlled trials;
(4) Observation index. The literature selection process is shown in Figure 1.

### 2.7 Literature quality evaluation

The two researchers independently assessed the risk of bias in the included literature by referring to the Cochrane system reviewer manual and using Review Management 5.3 software. The risk levels of literature quality bias are high, unclear and low. If there is a disagreement, the decision should be made in consultation with a third researcher.

### 2.8 Statistic analysis

#### 2.8.1 Data analysis and processing

The RevMan 5.3 software provided by the Cochrane Collaboration was used for statistical analysis. (1) For dichotomous variables, relative risk (RR) was used for statistics. For continuous variables, weighted mean difference (WMD) was selected when the tools and units of measurement indicators are the same. Standardized mean difference (SMD) was selected with different tools or units of measurement, and all the above were represented by effect value and 95% Confidence interval (CI). (2) Heterogeneity test: *Q* test was used to qualitatively determine inter-study heterogeneity. If *P* ≥ 0.1, there was no inter-study heterogeneity, If *P* < 0.1, it indicated inter-study heterogeneity. At the same time, *I*^2^ value was used to quantitatively evaluate the inter-study heterogeneity. If *I*^2^ ≤ 50%, the heterogeneity was considered to be good, and the fixed-effect model was adopted. If *I*^2^ > 50%, it was considered to have significant heterogeneity, the source of heterogeneity would be explored through subgroup analysis or sensitivity analysis. If there was no obvious clinical or methodological heterogeneity, it would be considered as statistical heterogeneity, and the random-effect model would be used for analysis. Descriptive analysis was used if there was significant clinical heterogeneity between the two groups and subgroup analysis was not available.

#### 2.8.2 Missing data processing

If data is missing or incomplete, we will contact the corresponding author to obtain the missing data. If not, this study will be removed.

#### 2.8.3 Heterogeneity and subgroup analysis

In order to eliminate the clinical heterogeneity between studies, subgroup analysis was conducted according to the types of western drugs and the course of disease.

#### 2.8.4 Sensitivity analysis

In order to test the stability of meta-analysis results of outcomes, a one-by-one elimination method will be adopted for sensitivity analysis.

#### 2.8.5 Reporting bias

For the major outcome indicators, if the included study was ≥10, funnel plot was used to qualitatively detect publication bias. Egger's and Begg's test are used to quantitatively assess potential publication bias.

#### 2.8.6 Evidence quality evaluation

The Grading of Recommendations Assessment, Development, and Evaluation (GRADE)^[23]^ will be used to assess the quality of evidence. It contains five domains (bias risk, consistency, directness, precision, and publication bias). And the quality of evidence will be rated as high, moderate, low, and very low.

## 3 Discussion

According to the clinical manifestation of pediatric adenoidal hypertrophy, Chinese medicine believes that the disease mostly belongs to the category of “HangSang,” “snoring sleep,” “sputum nucleus” and so on. Danxi Xinfa believes that the pathogenesis of adenoidal hypertrophied is based on deficiency in origin and excess in superficiality, because of children's delicate lung, the striae is not too solid to guard outside and defense, causing pathogenic factors coming into and stay the lung, inner heat builds up over time and finally burns the throat; Children's spleen is often insufficient, too much meat makes the spleen do not rise clear qi and transport irregularly, then wet accumulate into phlegm and store in the lung, gather in the nasopharynx. Adenoids are located in the nasopharynx, where the pharynx is the gateway to the lungs. Lung disease causes pharyngeal pain. The characteristics of this disease are: in the early stage, the insufficient lung and spleen, the pathogenic heat obstructing the lung are the main; then the hot blood unrestrained and coming into the collaterals over time; and in the later stage, the phlegm and coagulation blood stasis are the main. In the literature, Erchen Decoction and Shenling Baizhu powder are used to dry and invigorate the spleen in order to exhaust the source of phlegm. People with excessive lung heat obstructing are usually treated with Shengma Jiedu decoction, which is often combined with drugs such as Shengma (*Rhizoma Cimicifugae*), Huangqin (*Radix Scutellariae*), Pugongying (*Herba Taraxaci*) to clear heat and detoxify; Qi stagnation and blood stasis are often associated with Chishao (*Radix Paeoniae Rubra*), Taoren (*Semen Persicae*), Chuanxiong (*Rhizoma Chuanxiong*), Honghua (*Flos Carthami*), Danggui (*Radix Angelicae Sinensis*) to promoting blood circulation to remove blood stasis; In addition, targeted drugs will be added, such as Zaojiaoci (*Spina Gleditsiae*), Jiegeng (*Radix Platycodonis*) to release lung through removing pus, Xiakucao (*Spica Prunellae*), Kunbu (*Thallus Laminariae*), Zhebeimu (*Bulbus Fritillariae Thunbergii*) to remove and disperse phlegm knot; Children are the body of utmost Yang, Yang is often surplus, easy to become heat and harm Yin, in the late stage of the disease, Yin nourishing products are used, such as Shengdihuang (*Radix Rehmanniae*), Maidong (*Raidix Ophiopogonis*) to resist the warm property of xin medicine.

Taking Erchen Decoction as an example, modern pharmacological studies have shown that the essential volatile oil of Chenpi (*Pericarpium Citri Reticulatae*)^[5]^ has anti-asthma, anti-cough, anti-inflammatory and antibacterial effects, especially limonene^[6]^ has obvious inhibitory effects on pneumococcus, Streptococcus A and *Staphylococcus aureus*. Diisoprene bibenzyl in Gancao (Radix Glycyrrhizae) has an antiviral effect,^[7]^ and the flavone components in the leaves of Gancao have a strong antibacterial activity against gram-positive bacteria^[8]^; Poria cocos polysaccharide promotes lymphocyte proliferation and activation, inhibits inflammatory factors, and enhances immunity.^[9,10]^ Banxia (*Rhizoma Pinelliae*) has such effects as cough suppression, expectoration, anti-inflammatory and broad-spectrum antifungal, etc., stimulating adrenal glands to release glucocorticoids and producing glucocorticoid effect.^[11]^

Previous studies showed that the effective rates of montelukast or mometasone furoate nasal spray in the treatment of this disease were 62.4% and 63.1%, respectively.^[12]^ The new randomized controlled test shows that combined with Inhaled Corticosteroids (ICS) or Oral Montelukast Sodium (OM), its effective rate is 80%–90%,^[13]^ which is significantly higher than that of western medicine alone. Besides, the effect of TCM can be sustained and safe without appearing hormone dependence, drug withdrawal and recurrence. Therefore, it is necessary to carry out evidence evaluation on the treatment of adenoid hypertrophy with Chinese or western medicine, objectively evaluate the safety and clinical efficacy of this study, and provide feasible and effective treatment methods for the clinic. At the same time, this research has some limitations: literatures are only in Chinese and English, the included literatures are of low quality, allocation concealment and blinding implementation process is not clear, and follow-up time is short, the above factors can lead to a biased results, so we still need further study, finding the best way to explore the treatment of pediatric adenoidal hypertrophy.

## Author contributions

**Data collection:** Penglin Wang, Weidong Kong.

**Funding support:** Weidong Kong.

**Literature retrieval:** Yanchun Shan.

**Software operating:** Yanchun Shan.

**Supervision:** Yanchun Shan.

**Writing – original draft:** Penglin Wang, Weidong Kong.

**Writing – review & editing:** Penglin Wang, Weidong Kong.
